# The Impact of Hypertension and Atrial Fibrillation on Cognitive Decline and Subclinical Atherosclerosis

**DOI:** 10.3390/brainsci11060752

**Published:** 2021-06-06

**Authors:** Marius Militaru, Ciprian Rachieru, Daniel Florin Lighezan, Anda Gabriela Militaru

**Affiliations:** 1 Department of Neuroscience, Discipline of Neurology, Victor Babes University of Medicine and Pharmacy Timisoara, Municipal Emergency Hospital Timisoara, Piaţa Eftimie Murgu Nr. 2, 300041 Timisoara, Romania; 2 Department of Internal Medicine I, Discipline of Medical Semiology I, Victor Babes University of Medicine and Pharmacy Timisoara, Municipal Emergency Hospital Timisoara, Str. Ghe. Dima 5, 300079 Timisoara, Romania; ciprian.rachieru@umft.ro (C.R.); dlighezan@umft.ro (D.F.L.); militaru.anda@umft.ro (A.G.M.)

**Keywords:** cognitive impairment, hypertension, atrial fibrillation, subclinical atherosclerosis, memory tests

## Abstract

Background: Assessment of cognitive impairment and the presence of subclinical atherosclerosis are very important especially in patients with cardiovascular risk factors. Methods: We included 155 hypertensive patients (84 with AF versus 71 without AF) to identify the premature cognitive impairment, the earliest signs of subclinical atherosclerosis and onset of myocardial dysfunction and to evaluate the type of anticoagulation used, the importance of CHA₂DS₂-VASc score (</>3), age (</>65 years) in hypertensive patients with AF. Results: Mini-Mental State Examination (MMSE), Montreal Cognitive Assessment Scale (MoCA), Left Ventricular Ejection Fraction (LVEF) were significantly decreased, and Activities of Daily Living Score (ADL), Geriatric Depression Scale(GDS-15), and intima–media thickness (IMT) were significantly increased in hypertensive patients with AF vs. without AF (*p* < 0.05). MMSE was significantly decreased, ADL and IMT were significant increased in patients with AF and CHA₂DS₂-VASc>3 and non-vitamin K antagonists oral anticoagulants therapy (NOACs)(*p* < 0.05). Patients with age >65 with AF had higher rates of cognitive impairment (MMSE significant decrease) and a larger IMT (significant increase) versus patients with AF and age <65 (*p* < 0.05). Conclusions: Cognitive impairment is encountered in hypertensive patients having AF. Our conclusions suggest a direct link between cognitive impairment, depression, hypertension, AF, age, CHA₂DS₂-VASc score, type of anticoagulants used, LVEF, cognitive parameters, and IMT. We acknowledge the importance of identifying and preventing cognitive changes.

## 1. Introduction

Hypertension and atrial fibrillation (AF) are two important public health priorities. Hypertension is considered to be the strongest predictor of mortality all over the world [1]. The prevalence of hypertension is nowadays around 20% to 50% for the adult population globally [2]. Even if coronary heart disease and stroke mortality increase with modified blood pressure(BP) levels, specific cardiovascular events are differentin their relationship to BP [3].

Statistically, hypertension is considered the leading cause of cardiovascular disease that can be prevented and is one of the causes that lead to overall mortality worldwide and the prevalence can be over 10–15% in the elderly [4]. AF is considered to be one of the most common cardiac arrhythmias in the world among adults. In the general population, the prevalence is about 2–4%, the estimates showing an increase in the next years of 2.5 times due to the increase of life expectancy, but also, to the intensification of the diagnostic methods [5]. Hypertension is the most common etiological factor associated with the development of an episode of AF, there being a very close relationship between the two factors. The etiopathogenic relationship between hypertension and AF is explained primarily by left ventricular (LV)—printed diastolic dysfunction and consecutive left atrial remodeling [4,5]. Hypertension can cause changes in inflammation and fibrosis of the left atrium (LA). These changes can develop hypertrophy of the LA, which results in the development of AF. It is known that the hemodynamic mechanism is most involved in AF. It consists of an increase in the thickness of the LV walls and of the LV diastolic dysfunction. Through these changes, remodeling dysfunction of the LA favors the appearance of AF [4,5].The combination of hypertension and AF may increase the risk of stroke or heart failure, but patients suffering from those may also die faster compared to patients having no AF [6].

Another determining factor of the degradation of cerebral functions, although less clinically obvious, is the destruction of intracerebral microcirculation as well as cerebral vascular microembolism in the conditions of inconsistent control of blood pressure values [4]. Moreover, there is a controversy, for example, between the optimal blood pressure reference values proposed by the European Society of Cardiology (ESC) 2018 guide [4] and the American College of Cardiology/American Heart Association (ACC/AHA) 2017 guide [7], including regarding the blood pressure control of the elderly, but also of those with multiplecomorbidities [4,7].Risk tripod—diabetes, hypertension, dyslipidemia, along with smoking, are significant methods of risk assessment [4]. Moreover, the mode of management of thromboembolic risk in AF-type of anticoagulation, rhythm/frequency control, hemodynamics, application of thrombotic risk scoresCHA2DS2-VASc, more recently ABC, respectively hemorrhagicHAS-BLED, are also contributors [5]. In addition, the evolution of atherosclerosis but also of arteriosclerosis on a hypertensive and dysmetabolic background is independent of the systemic embolicrisk, and it must be monitored and influenced as much as possible, by new therapeutic targets and by new molecules with evidence of efficacy and tolerability in monitoring dyslipidemia and diabetes [4,5,7].

The identification of cardiovascular risk factors, the application of risk scores, and the analysis and calculation of ultrasound parameters can lead to the prevention of complications of hypertension and AF and thus to the prevention of cerebral thromboembolic events or in other systemic territories.

Hypertension and dementia are frequently observed in the general population [2]. Hypertension has been shown to be a risk factor for cognitive impairment, Alzheimer’s disease (AD), and vascular dementia (VD) [8]. 

Specialized literature shows that there is a close link between AF and stroke in regard to the occurrence of cognitive impairment [9]. There are studies that have shown the relationship between AF and Alzheimer’s dementia independent of stroke incidence [10]. 

The increase in the number of elderly patients having AF also has a result in the appearance and evolution of cognitive disorders and affecting their quality of life [11]. 

Effective oral anticoagulation and improved management of the overall cardiovascular risk profile in people with AF offer the promise of reducing the impact of AF on cognitive impairment and dementia [11]. 

In order to establish the diagnosis of cognitive disorder and depression and to evaluate the daily activity of the patient, rigorous clinical analysis and the performance and interpretation of the scores of screening tools for cognitive impairment, daily activity, and depression tests are needed.

Depression is a neuropsychiatric disorder that is often associated with cognitive impairment. The presence of depression can change the clinical condition of patients with cognitive impairment and thus can influence their daily activity. There have been several studies [12] that have shown that depression is a risk factor for the progression of cognitive impairment. In major depression, cognitive impairment is documented and it is part of the Diagnostic and Statistical Manual (DSM) criteria for depression [12,13]. If episodes of AF are more frequent, depression increases, and it is associated with the severity of symptoms, increased mortality, and with the increased need for medical care [13]. 

In this study we analyzed whether there is a close relationship between cognitive impairment, according to the fifth edition of the American Psychiatric Association’s Diagnostic and Statistical Manual (DSM-5) criteria [14], hypertension and AF, regardless of the presence of stroke by analyzing screening tools for cognitive impairment, daily activity and depression scales, subclinical atherosclerosis, and cardiac performance. For example, scanning by vascular ultrasound but especially by Angio computed tomography(CT) of the arterial tree, with the highlighting of atherosclerotic subclinical lesions can bring contributing data [4,7]. Not without importance is the non-invasive assessment of endothelial dysfunction through the use of vascular ultrasound [4,7].

On the other hand, it is necessary for a more adequate detection of paroxysmal or asymptomatic AF accesses, through personal devices—smartphones, etc., or outpatient Holter exams, telemetry, etc. [5]. Systemic hemodynamics need to be monitored, impaired systolic function, an increase in left atrium volume as well as the appearance of thrombosis in the left atrium appendage or a spontaneous contrast effect may be very helpful in assessing risk [5].

There is a need for a better understanding of the factors that lead to cognitive impairment among the patients with AF and hypertension, as well as knowledge of the potential mechanisms that explain such associations, all this being necessary to develop strategies of AF management, prevent the occurrence of cognitive impairment and improve quality of life among these patients.

The purpose of this study is to assess the cognitive impairment, depression, and daily activity using screening tools for cognitive impairment, (Mini-Mental State Examination (MMSE), Montreal Cognitive Assessment (MoCA)), daily activity scales (Activities of Daily Living Score (ADL), Instrumental Activities of Daily Living Score (IADL)) and depression scale, Geriatric Depression Scale (GDS-15), the occurrence of subclinical atherosclerosis using arterial parameters IMT and ankle brachial index(ABI) and the onset of myocardial dysfunction using echocardiographic parameters of left ventricle function (LVEF), and the association between subclinical atherosclerosis and cognitive impairment in hypertensive patients with AF vs. hypertensive patients without AF. Also, we want to evaluate the importance of age (less or more than 65 years old), CHA₂DS₂-VASc score, and the role of anticoagulation(oral anticoagulants using vitamin K antagonists (VKAs) therapy (OACs) or NOACs), in hypertensive patients with AF on cognitive impairment and signs of subclinical atherosclerosis.

## 2. Materials and Methods

### 2.1. Study Population and Clinical Assessment

We included 155 hypertensive patients with/without AF in the study. We compared the results from the cognitive impairment screening and subclinical signs of atherosclerosis of the 84 patients with AF (paroxysmal, persistent, or permanent) versus 71 patients without AF. The patients were hospitalized in internal medicine or neurology clinics. The presence of hypertension and other cardiovascular risk factors with/without AF and patients with absence of a record of cognitive impairment or dementia according to DSM-5 criteria [14], with OACs or NOACs therapy were the inclusion criteria. Hypertension was defined as a persistent elevation in office systolic BP ≥ 140 and/or diastolic BP ≥ 90 mmHg (constant, repeated, with patients seated quietly for 5 min before determinations)and/or taking of antihypertensive medication. Hyperlipemiawas defined as abnormally elevated levels of lipids or taking of lipid-lowering medication. The cut-off of hyperlipemia was as follows: total cholesterol(TC) concentration ≥200 mg/dL, low-density lipoproteincholesterol (LDL-C) ≥130 mg/dL, triglycerides(TG)>150 mg/dL and high-density lipoprotein cholesterol (HDL-C)<35 mg/dL. Smoking was evaluated using the pack-year, a unit for measuring the amount a person has smoked over a long period of time, for example, 1 pack-year is equal to smoking 20 cigarettes (1 pack) per day for 1 year. We included patients who have smoked more than 10 cigarettes a day for 10 years (smoking history more than 5 pack-year history). Obesity was defined after body mass index (BMI) and clinical evaluation.Chronic kidney disease (CKD) was defined as decreased kidney function shown by glomerular filtration rate (GFR) of less than 60 mL/min per 1.73 m^2^, or markers of kidney damage, or both, of at least 3 months duration, regardless of the underlying cause, and/or according to the patients’ medical history.Peripheral arterial disease (PAD) was defined as an ABI less than 0.9 and/or according to the patients’ medical history [4,5,7].Coronary disease, heart failure, and lacunar or minor stroke were analyzed according to the patients’ medical history.AF was determined according to electrocardiography at rest and cardiac physical examination results, independently of its paroxysmal, persistent, or permanent nature. A strong limitation of the study was that cranial CT could not be performed for the patients included in the study. All standards in regard to good clinical practice and ethics were observed during our study. All the patients have undergone clinical and biochemical analyses, screening tools for cognitive impairment, daily activity, and depression scales including MMSE, MoCA, ADL, IADL, and GDS -15 and echocardiographic evaluation to identify the earliest signs of subclinical atherosclerosis and hemodynamic changes.

Clinical assessment, done for all patients included in the study, consisted of: medical history, clinical examination, registration of systolic blood pressure (SBP), diastolic blood pressure (DBP), and heart rate (HR). Paraclinical evaluation contained: blood tests, resting electrocardiogram (EKG), Doppler carotid artery ultrasound with the evaluation of arterial parameters of subclinical atherosclerosis IMT, evaluation of ABI and transthoracic echocardiography, with evaluation of left ventricular parameters function. The CHA₂DS₂-VASc score was calculated for all patients suffering from AF.

### 2.2. Parameters of Subclinical Atherosclerosis

#### 2.2.1. Intima–Media Thickness (IMT)

A General Electric Vivid E9 ultrasound system with a 9L MHz transducer was used to calculate the IMT value. It was calculated at the distal wall of the common carotid artery (CCA), 1 cm from the carotid bulb. For each patient, we registered ten measurements and we calculated the mean values. All carotid echography examinations were performed on the same echograph and by the same examiner.

#### 2.2.2. Ankle Brachial Index (ABI)

ABI is the ratio of the blood pressure at theankleto the blood pressure in the upperarm(brachium). The ABI test is a quick, noninvasive way to check for peripheral artery disease (PAD). A low ABI score can indicate a narrowing of the arteries that reduce blood flow in the legs.An ABI between 1.0 to 1.4 range suggests that the patient doesn’t havePAD, an ABI between 0.91 to 0.99range indicates that the patient has borderlinePADand an ABIless than 0.90is considered abnormal and indicates a diagnosis of PAD [15]. 

### 2.3. Transthoracic Echocardiography Left Ventricular Parameters Function

A General Electric Vivid E9 device was used to perform transthoracic cardiac ultrasound, measurements and evaluations were performed using an M5S MHz transducer. All cardiac ultrasound evaluations were performed by the two-dimensional 2D method. LV systolic function was assessed by calculating the left ventricular ejection fraction (LVEF) by the planimetric method. The following formula for determining LVEF was used using left ventricular diastolic diameter (LVEDD) and left ventricular systolic diameter (LVESD): LVEF = (LVEDD–LVESD)/LVEDD × 100. LV diastolic function was also assessed. The color Doppler and then the pulsed Doppler (PW) were superimposed and the E wave of the transmittal flow was measured, this representing the peak early diastolic transmitral flow velocity. We would like to mention that the same echocardiograph was used, and the transthoracic echocardiographic examinations were performed by a single examiner.

### 2.4. Screening Tools for Cognitive Impairment, Daily Activity, and Depression Scales

#### 2.4.1. Mini-Mental State Examination (MMSE)

MMSE examination was used in screening for cognitive impairment. MMSE is a scale used to assess cognitive functions such as attention, calculation, orientation, recall, construction practices, and language manipulation [16,17,18]. It is used to assess the progression and severity of cognitive impairment and also to analyze a patient’s cognitive changes over time. This score must be analyzed according to the level of education and age [16,17]. Score 30 is the maximum score, and a score of 23 is representative of cognitive impairment and dementia [16,18]. 

#### 2.4.2. Montreal Cognitive Assessment (MoCA)

The MoCA scale is extensively used to evaluate cognitive impairment. It is a scale with a maximum score of 30 points that evaluates several cognitive functions: orientation, visuospatial ability, language, attention, concentration, working memory, short-term memory recall task, executive functions, a three-dimensional cube copy, a phonemic fluency task, a two-item verbal abstraction task. The duration of the test is approximately 10 min and a score below 26 means the occurrence of cognitive impairment and dementia [19]. 

#### 2.4.3. Activities of Daily Living (ADL)

The ADL score is the scale used in the area of healthcare to a high extent to refer to the day-to-day care activities of the patient. ADL is a scale used as an indicator of a person’s functionality status. Using this scale, we can describe a person’s ability to care for themselves independently. The ADL scale analyzes a person’s abilities to perform the following six physical needs: dressing, bathing, transferring, toileting, continence, feeding. The inability of a person to perform essential activities of daily living can lead to decreased quality of life and the need to depend on other people [20]. 

#### 2.4.4. Instrumental Activities of Daily Living (IADL)

The IADL scale is used especially in older patients for daily practical activities to assess the ability to function and daily care and uses eight areas to evaluate more complex activities necessary for normal functioning such as financial management, cooking, or shopping, with a minimum score of 0 which represents low functionality and a maximum score of 8 which represents high functionality. The IADL scale is used by questions addressed to the patient or by a written form completed by the patient [21]. 

#### 2.4.5. Geriatric Depression Scale (GDS-15)

This scale is used to assess depression in populations, especially in elderly patients. For an easier evaluation, the short scale with 15 questions (GDS-15) is used more frequently, and also used in our study, this being easier to use both in patients without cognitive impairment and in those with cognitive impairment where an evaluation with several questions would be difficult [22]. GDS-15 is a short questionnaire with a specificity of 89% and a sensitivity of 92% that lasts approximately 10–15min in which participants are asked to answer the 15 questions and the scores obtained indicate or not diagnostic elements of depression [22,23]. 

### 2.5. CHA₂DS₂-VASc Score for Atrial Fibrillation Stroke Risk

CHA₂DS₂-VASc Score is used to evaluate AF stroke risk. The score is: Congestive heart failure 0 or 1, Hypertension 0 or 1, Age 75 years or older 0 or 2, Diabetes mellitus 0 or 1, Stroke/Thromboembolism 0 or 2, Vascular disease 0 or 1, Age 65 to 74 years 0 or 1 and sex (female gender confers higher risk). A highscorecorresponds to a greater risk of stroke, while a lowscorecorresponds to a lower risk of stroke. TheCHA₂DS₂-VAScscorehas been validated by many studies [24]. 

### 2.6. Statistical Analysis

IBM SPSS program version 20.0 was performed for statistical analysis. Results are expressed as mean value ± standard deviation, percentages. Evaluation of blood tests, HR, SBP, DBP, LV function parameters, IMT, ABI, memory, the activity and instrumental activity of daily living, and depression tests were performed using unpaired T-test, in both groups of patients. The use of OACs or NOACs, CHA₂DS₂-VASc score and evaluation by age <65 years old or >65 years old in hypertensive and AF patients were calculated using unpaired T-test. Analyses of covariance (ANCOVA) were used to investigate the effects of IMT values and screening tools (MMSE, MoCA, ADL, IADL, and GDS-15) on hypertensive patients with AF and hypertensive patients without AF with control for age and sex. The values for *p* < 0.05 and *p* < 0.001 were considered statistically significant.

## 3. Results

### 3.1. Study Population

All 155 hypertension patients that we studied had a mean age of 69.98 ± 9.89 (44–89 years). Among them 83 (53.5%) were females and 72 (46.5%) were men;110 were older than 65 years old (71%) and 45 (29%) patients were younger than 65 years old.

Among all 155 hypertensive patients, 24 (15.5%) were with hypertension grade I, 72 (46.5%) with hypertension grade II, and 59 (38.1%) with hypertension grade III.

Among all hypertensive patients: 83 (53.5%) were with hyperlipemia, 49 (31.6%) were smoking patients, 46 (29.7%) were with diabetes mellitus, and 25 (16.1%) with obesity (Table 1). There were84 (54.2%) hypertensive patients with AF that had a mean age of 73.88 ± 9.78 (48–89 years). There were 71 (45.8%) hypertensive patients without AF and thathad a mean age of 68.00 ± 9.23 (44–88 years)(Table 1).

Ofthe 84 hypertensive patients with AF, 16 (19%) were with persistent AF, 29 (34.6%) were with paroxysmal AF and 39 (46.4%) were with permanent AF. Ofthe patients with AF, 10(11.9%) were with hypertension grade I, 42(50%) with hypertension grade II, and 32(38.1%) with hypertension grade III.

The complete list of patients’ characteristics is reported in Table 1.

### 3.2. Comparative Analysis of Biologic Parameters in Hypertensive Patients with/without AF

Hemoglobin (Hb)(g/dL)and thrombocytes (×10^3^/µL) were statistically significantly lower in hypertensive patients with AF, compared to hypertensive patients without AF, but without values within pathological limits (*p* < 0.05), and activated partial thromboplastin time(APTT)(s) was statistically significantly higher in patients with AF, 28.87 ± 7.97, compared to those without AF, 26.41 ± 4.46 (*p* < 0.05), possibly due to the presence of anticoagulant treatment in patients with AF.

The decrease in Hb and thrombocytes and increase in APTT in patients with AF may be due to the presence of anticoagulant therapy in these patients.

Regarding the impairment of the lipid profile, it was shown that there are statistically significantly lower values of total cholesterol and low-density lipoprotein(LDL) cholesterol in hypertensive patients with AF than those without AF, without pathological values of lipid profile (*p* < 0.05). This means that there is better control of lipid profile values through diet and treatment in patients with hypertensive AF. The other components of the lipid profile (high-density lipoprotein(HDL) cholesterol and triglycerides(TG)) did not show statistically significant differences in the two groups of patients.

Creatinine (mg/dL) was statistically significantly higher in hypertensive patients with AF, 1.04 ± 0.23, compared to those without AF, 0.91 ± 0.23 (*p* < 0.05) and transaminases (alanine aminotransferase (ALT), aspartate aminotransferase(AST)) showed statistically significantly higher values in hypertensive patients with AF, ALT (U/L) 32.77 ± 26.23, respectively AST (U/L) 29.22 ± 22.91, compared to patients without AF, ALT 26.49 ± 10.44, respectively AST 22.30 ± 6.97 (*p* < 0.05), without presenting pathological values in the two groups of patients.

When evaluating the other biological parameters, no statistical changes were highlighted in the two groups of patients. The complete list of all the characteristics of the biological parameters is reported in Table 2.

### 3.3. IMT, ABI, and Hemodynamic Parameters (SBP, DPB, HR) in Both Groups of Patients

IMT (mm) measured on left CCA and on right CCA were statistically significantly higher in hypertensive patients with AF, IMT (mm) left 0.75 ± 0.27, and IMT (mm) right 0.75 ± 0.32, compared to hypertensive patients without AF, IMT (mm) left 0.75 ± 0.32, and IMT (mm) right 0.59 ± 0.26 (*p* < 0.05).

Regarding the evaluation of ABI, there were no statistically significant changes in the two groups of patients, but the values were slightly lower in hypertensive patients without AF and did not show the pathological significance of PAD.

Regarding hemodynamic parameters, BP (mmHg) was statistically significantly higher in hypertensive patients with AF, SBP (mmHg) 136.31 ± 22.61, respectively DBP (mmHg) 80.71 ± 14.16, compared to hypertensive patients without AF, SBP (mmHg)127.04 ± 14.96 and respectively, DBP (mmHg) 75.14 ± 14.01 (*p* < 0.05). These changes in hemodynamic parameters and IMT mean that BP is higher in patients with AF and that the process of subclinical atherosclerosis is evident in this group of patients. These increases in SBP and DBP in patients with AF may be due to a longer history of hypertension than in patients without AF.

The complete list of all hemodynamic and subclinical atherosclerosis parameters is reported in Table 3.

### 3.4. Comparative Analysis of Echocardiographic Parameters of Left Ventricle Systolic Function in Both Groups of Patients

The analysis of echocardiographic parameters in the 2D sequence showed that left atrial, LA (mm), was statistically significantly higher 43.25 ± 6.50 in hypertensive patients with AF compared to 39.15 ± 5.15 in hypertensive patients having no AF (*p* < 0.05). Echocardiographic parameters LVEDD (mm) and pulmonary systolic arterial pressure(PSAP)(mmHg) were statistically significantly higher in hypertensive patients having AF than in hypertensive patients having no AF (*p* < 0.05).

LVEF (%) was statistically significantly lower 56.29 ± 7.77 in hypertensive patients with AF compared to 58.95 ± 5.45 in hypertensive patients without AF (*p* < 0.05). The LV diastolic dysfunction parameters and other parameters obtained by 2D cardiac ultrasound did not change statistically in hypertensive patients with AF compared to hypertensive patients without AF.From the all 84 hypertensive patients with AF, 15 (17.9%) patients registered LVEF <50% and from the 71 hypertensive patients without AF, 3 (4.2%) patients registered LVEF <50%.

The analysis of echocardiographic parameters is exemplified in Table 4.

### 3.5. Comparative Analysis of Screening Tools for Cognitive Impairment, Daily Activity, and Depression Scalesin Both Groups of Patients

Of the 84 hypertensive patients with AF evaluated in the study, 42 (50%) patients had an MMSE score above 27, 16 (19%) patients had an MMSE score between 24 and 27 and 24 (31%) patients had an MMSE score of less than 24.

Of the 71 hypertensive patients without AF introduced in the study, the number of hypertensive patients without AF in terms of the presence of cognitive impairment was lower, so there were 7 (9.9%) patients with MMSE had a score that ranged from 24 to 27, 4 (5.6%) patients with MMSE score less than 24, the remaining 60 (84.5%) patients with an MMSE score higher than 27, without the presence of cognitive impairment.

Evaluation of parameters using screening tools for cognitive impairment, daily activity, and depression scalesshowed: a statistically significant decrease in cognitive parameters (MMSE (*p* < 0.001) and MoCA(*p* < 0.05)) as well as an increase in parameters that assess the daily activity of patients (ADL, IADL)(*p* < 0.05), and the parameter for assessing the degree of depression (GDS-15)(*p* < 0.05) of hypertensive patients having AF compared to hypertensive patients without AF.

Regarding sex, MMSE was statistically significantly lower 25.21 ± 5.10 in hypertensive female patients with AF, compared with hypertensive patients without AF 28.26 ± 2.90 (*p* < 0.05). MoCA was also statistically significantly lower, GDS-15, and SBP (mmHg) were statistically significantly higher in female patients with AF than those without AF (*p* < 0.05). IMT (mmHg) was statistically significantly higher in both male and female hypertensive patients with AF than those without AF (*p* < 0.05).

This means that hypertensive patients with associated AF may have a greater degree of cognitive impairment, slightly altered daily activity, and associated depression, compared to hypertensive patients without AF. These aspects are more representative for patients who have hypertension and AF as well as other associated risk factors and other associated pathologies for a long time and who already present signs of cognitive impairment, regardless of gender.

The results of all screening tools for cognitive impairment, daily activity, and depression scalescores are exemplified in Table 5.

### 3.6. Comparative Analysis of Types of Anticoagulants Used in Hypertensive Patients with AF Patients on Screening Tools for Cognitive Impairment, Daily Activity, and Depression Scales, Subclinical Atherosclerosis, Hemodynamic and Echocardiographic Parameters

All patients with AF were under anticoagulant therapy. Of the 84 patients with AF, there were 31 (36.9%) patients treated with non-vitamin K oral antagonist anticoagulant therapy (NOACs) and 53 (63.1%) patients treated with oral anticoagulants using vitamin K antagonists (VKAs)—acenocoumarol therapy—(OACs). Of those 31 patients on NOACs, 14 (16.7%) on dabigatran (direct thrombin inhibitor) therapy, 12 (14.3%) were on apixaban (direct factor Xa inhibitor) therapy, and 6 (7.1%) on rivaroxaban (direct factor Xa inhibitor) therapy.

**Figure 1:**
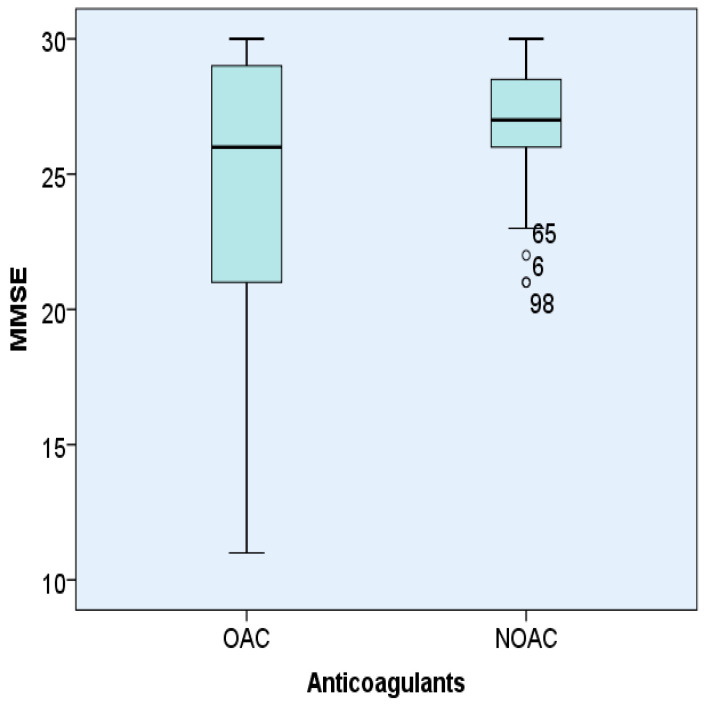
Representation of MMSE according to the type of anticoagulant (OACs orNOACs) used in hypertensive patients with AF.

MMSE was statistically significantly lower in patients with AF treated with OACs 24.30 ± 5.75 compared to patients treated with NOACs 26.61 ± 2.52 (*p* < 0.05) (Figure 1).

Evaluation by screening tools for cognitive impairment, daily activity, and depression scalesof the other parameters (MoCA, ADL, IADL, GDS-15), as well as of the subclinical atherosclerosis parameters, hemodynamic and echocardiographic parameters did not bring statistically significant changes regarding the use of NOACs or OACs treatment. This means that some of the screening tools for cognitive impairment, daily activity, and depression scales, as well as the echocardiographic, hemodynamic, and subclinical atherosclerosis parameters are not influenced by the type of anticoagulant used.

The MMSE score showed that the use of NOACs could explain better cognitive control of hypertensive patients with AF. The other parameters, MoCA was lower, ADL, IADL, and GDS-15 were higher in patients treated with OACs than in those treated with NOACs, but statistically insignificant.

Analysis of the MMSE score in patients with AF and hypertension depending on the type of anticoagulant used (OACs or NOACs). The box represents the interquartile range, the line represents the median value and the bars represent the minimum and maximum values. Points outside the boxplot represent outlier values.

### 3.7. Comparative Analysis of CHA₂DS₂-VASc Score (</>3) in Patients with AF on Screening Tools for Cognitive Impairment, Daily Activity, and Depression Scales, Subclinical Atherosclerosis, Hemodynamic and Echocardiographic Parameters

The 84 patients with hypertension and AF introduced in the study had a CHA₂DS₂-VASc score of 3.55 ± 1.40 between 1 and 7.21 (25%) patients with AF had a CHA₂DS₂-VASc score of < 3, and 63 (75%) AF patients had a CHA₂DS₂-VASc of score >3.

Of the patients with a CHA₂DS₂-VASc score of < 3, two patients had an MMSE score less than 24, one patient had an MMSE score between 24 and 27, and the other 18 patients had an MMSE score above 27. With a score of CHA₂DS₂-VASc >3 were 24 patients with an MMSE score less than 24, 15 patients with an MMSE score between 24 and 27, and the other 24 patients hadan MMSE score above 24.

The number of patients with an MMSE score less than 24 or between 24 and 27 was higher among hypertensive patients with AF and the presence of risk factors and associated vascular pathology according to the CHA₂DS₂-VASc score.

MMSE was statistically significantly lower in hypertensive patients with AF with a CHA₂DS₂-VASc score > 3, 24.27 ± 5.28, compared with patients with AF with a CHA₂DS₂-VASc score < 3, 27.81 ± 2.04 (*p* < 0.001).MoCA was statistically significantly lower in hypertensive patients with AF with a CHA₂DS₂-VASc score > 3, 22.32 ± 5.73, compared with patients with AF with a CHA₂DS₂-VASc score < 3, 26.81 ± 2.78 (*p* < 0.001).

**Figure 2:**
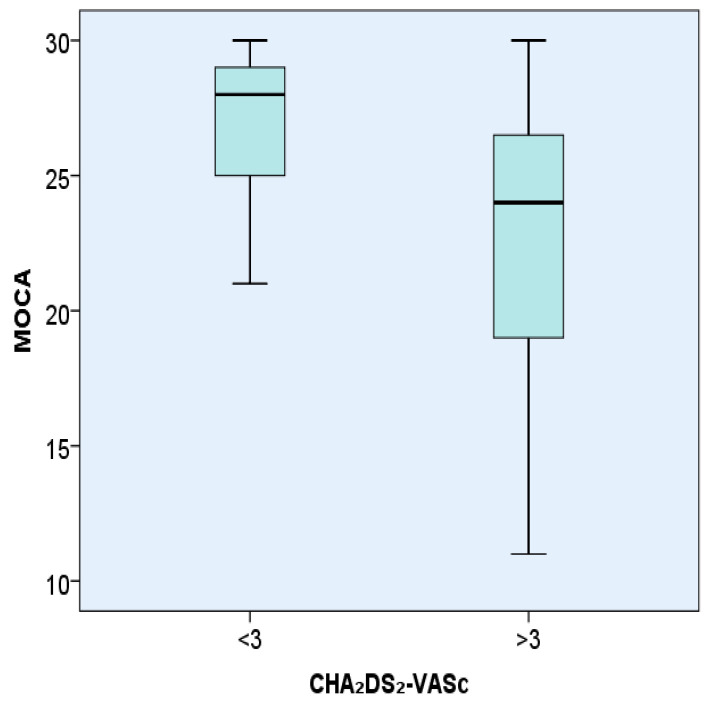
Representation of the MoCA test in hypertensive patients with AF according to the CHA₂DS₂-VASc score </> 3.

The representation of MoCA test values in hypertensive patients with AF introduced in the study according to the CHA₂DS₂-VASc score </>3 is shown in Figure 2.

Analysis of the MoCA score in patients having AF and hypertension according to the CHA₂DS₂-VASc score (</>3). The box is a representation of the interquartile range, the line represents the median value and the bars represent the minimum and maximum values.

Regarding the analysis of the other parameters according to the CHA₂DS₂-VASc score (</> 3), it was highlighted that the parameters that evaluate the daily activity of patients (ADL, IADL), as well as the parameter on the degree of depression (GDS-15), were statistically significantly higher in patients with AF and CHA₂DS₂-VASc score > 3 than in patients with AF and CHA₂DS₂-VASc score < 3, (*p* < 0.05).

The scores of all screening tools for cognitive impairment, daily activity, and depression scalesare reported in Table 6.

The values of IMT (mm) left CCA 0.77 ± 0.28 and IMT right CCA 0.79 ± 0.34 were statistically significantly higher in patients with AF and CHA₂DS₂-VASc score > 3 compared to the values of IMT (mm) left CCA 0.68 ± 0.21, respectively, IMT (mm) right CCA 0.62 ± 0.24 in patients with AF and CHA₂DS₂-VASc score < 3(*p* < 0.05).

With the other hemodynamic, echocardiographic, and ABI parameters, no statistically significant changes were highlighted according to the CHA₂DS₂-VASc score, but both LVEF (%) and ABI scores were lower in patients with AF and CHA₂DS₂-VASc score > 3.

The presence of lower scores of cognitive assessment parameters, as well as higher scores of parameters of daily activity, depression, and IMT, on the signs of subclinical atherosclerosis, are justified by the increase in the number of risk factors and associated vascular pathology in hypertensive patients with AF and higher CHA₂DS₂-VASc score; therefore, the more a hypertensive patient associates AF and other cardiovascular risk factors and associated vascular pathology, there is a greater chance they may have a more pronounced cognitive impairment, daily activity disorders, elements of depression as well as signs of subclinical, or even clinically manifest, atherosclerosis.

### 3.8. Comparative Analysis of Age </>65 in Hypertensive Patients with AF on Screening Tools for Cognitive Impairment, Daily Activity, and Depression Scales, Subclinical Atherosclerosis, Hemodynamic and Echocardiographic Parameters

Of the 84 hypertensive patients with AF aged between 49 and 89 years, there were 18 (21.4%) aged under 65 years and 66 (78.6%) aged over 65 years.

MMSE was statistically significantly lower 24.53 ± 5.15 in patients with AF and age >65 years compared to 27.44 ± 2.14 in patients with AF and age <65 years. IMT—CCA right was statistically significantly higher 0.79 ± 0.31 in hypertensive patients with AF aged >65 years compared to 0.59 ± 0.33 in hypertensive patients with AF aged <65 years.

Of the 110 patients over the age of 65, 66 (60%) were patients with AF, and 44 (40%) were patients without AF. MMSE was statistically significantly lower, and GDS-15, SBP, DBP, and IMT were statistically significantly higher in patients over 65 years of age with hypertension with AF than those with hypertension without AF (*p* < 0.05).

The other parameters evaluated using screening tools for cognitive impairment, daily activity, depression scales, hemodynamic and ultrasound tests showed more pronounced changes in hypertensive patients with AF and age >65 years, but there were no statistically significant changes in the two groups of patients.

These results show that hypertensive patients with AF over the age of 65, have a higher risk of developing cognitive impairment, as well as a greater increase in signs of subclinical atherosclerosis and the occurrence of cardiovascular or cerebrovascular events.

### 3.9. IMT and Screening Tools For Cognitive Impairment, Daily Activity, and Depression Scalesin Both Groups of Patients Adjusted for Age and Sex

Analyzing IMT values (right and left) by groups of hypertensive patients with AF and hypertensive patients without AF, we obtained that the highest values were observed in hypertensive patients with AF, these values being significantly higher compared to those recorded in hypertensive patients without AF (Table 3). The differences remained statistically significant after adjusting by age (Table 7). However, the difference was not found as statistically significant after adjusting by sex (Table 7).

Analyzing MMSE and MoCA scores by groups of hypertensive patients with AF and hypertensive patients without AF, we obtained that the lower values were observed in hypertensive patients with AF, these values being significantly lower compared to those recorded in hypertensive patients without AF (Table 5). The differences remained statistically significant after adjusting by age (Table 7). However, the difference was not found as statistically significant after adjusting by sex (Table 7).

Similarly, analyzing ADL, IADL, and GDS-15 scores by groups of hypertensive patients with AF and hypertensive patients without AF, we obtained for each, that the higher values were observed in hypertensive patients with AF, these values being significantly higher compared to those recorded in hypertensive patients without AF (Table 5). The differences remained statistically significant after adjusting by age (Table 7). However, the difference was not found as statistically significant after adjusting by sex (Table 7).

## 4. Discussion

High blood pressure and AF are two important public health priorities. Hypertension and AF are responsible for a very high risk of cardiovascular disease and death. Hypertension is the most important modifiable risk factor for AF. Worldwide, their prevalence is increasing, and we often meet them at the same time in the same patient [25]. It is not very well explained whether the relationship between BP and the risk of AF is linear or whether there is a certain value of BP over which the risk of AF increases [26]. Ofall 155 hypertensive patients, 24 (15.5%) were with grade I hypertension, 72 (46.5%) with grade II hypertension, and 59 (38.1%) with grade III hypertension. Of all patients included in the study, 84 (54.2%), just over 50%, had associated hypertension and AF. In specialized studies, it is estimated that hypertension is present in about 60% to 80% of patients with AF [25]. 

Of the 84 hypertensive patients with AF from the study, 10 (11.9%) were with grade I hypertension, 42 (50%) with grade II hypertension, and 32 (38.1%) with grade III hypertension. High blood pressure increases the risk of AF and, due to its high prevalence in the population, it presents a higher risk to produce more cases of AF than other risk factors. In a case-control population study, the risk of AF doubled in people with SBP ≥ 150 mmHg, compared to patients with SBP between 120 and 129 mmHg. There was an increased risk of AF in those with SBP < 120 mmHg, consistent with the J curve phenomenon while this excess risk was not significant in patients with SBP between 140 and 149 mmHg [27]. Better control of BP in hypertensive patients could reduce the risk of developing AF. In our study, both DBP (mmHg) and SBP (mmHg) were statistically significantly higher in hypertensive patients with AF than in hypertensive patients without AF (*p* < 0.05), DBP (mmHg) by about 5 mmHg and SBP (mmHg) with approximately 10 mmHg. Experimental studies have shown that high blood pressure can be a cause of AF because it can develop changes in inflammation, fibrosis, and hypertrophy of the LA. These studies have shown that there are architectural, structural, and electrophysiology changes. The most common mechanism seems to be the hemodynamic one, which is produced by increasing the thickness of the LV wall, there is an increase in LV stiffness, and thus occurs the diastolic LV dysfunction associated with hypertension. These mechanisms determine at the level of LA, remodeling phenomena with LA dysfunction which predisposes to the appearance of AF. The results of these studies have shown that if better control of blood pressure in hypertensive patients is performed, these structural modifications of the LA can be prevented, and thus the development of AF can be stopped [25]. 

A meta-analysis of 18 randomized studies, which compared a higher target value of BP with a lower one, showed that the higher target significantly reduced the risk of heart failure (HF) by 25%, myocardial infarction by 15%, stroke by 20%, and cardiovascular death by 18% [28]. In our study, the HR (b/min) was higher in hypertensive patients with AF, regardless of its type (paroxysmal, persistent, or permanent), than in those without AF, but statistically insignificant.

In our study, creatinine (mg/dL), APTT, and transaminases were statistically significantly higher in hypertensive patients with AF, compared to those without AF, (*p* < 0.05). The increase in creatinine in patients with AF can be explained by the presence in the study of a double number of patients with chronic kidney disease and AF compared to those without AF.

Regarding biological parameters, the limitations of the study are these changes in APTT levels because we do not have a big sample size of anticoagulant patients to have good stratification of our population, and also, creatinine levels and transaminase levels were not stratified for comorbidities, concomitant drugs, sex, and age.

In our study, regarding the evaluation of the lipid profile according to hypertension and the presence of AF, it was shown that there are statistically significantly lower values of total cholesterol and LDL cholesterol in hypertensive patients with AF than those without AF (*p* < 0.05). A study that looked at the relationship between lipid profile and the presence of paroxysmal AF showed that the relative risk of paroxysmal AF for patients with low total cholesterol, TG, and HDL cholesterol was 4.08 (95% CI: 1.81−9.57) times and 9.40 (3.25–32.0) times higher (*p* < 0.01), compared to patients with high total cholesterol, TG respectively, HDL cholesterol [29]. A study evaluating 651 control patients with paroxysmal or permanent AF revealed that patients with AF had significantly higher TG levels and lower levels of LDL cholesterol and HDL cholesterol (*p* < 0.05). Compared to control patients, those with AF had lower levels of HDL cholesterol and LDL cholesterol. TG (OR 0.945, *p* < 0.66496) and total cholesterol (OR 0.979, *p* < 0.9247) were negatively and linearly associated with paroxysmal AF, while HDL cholesterol (OR 0.136, *p* = 0.0002), LDL cholesterol (OR 0.334, *p* = 0.0036) and TG (OR 0.807, *p* = 0.2042) were negatively and linearly associated with continuous AF [30]. The presence of a lower lipid profile in patients with AF in our study can be explained as in other studies, which showed that adequate BP control and adequate dietary intervention with a low lipid profile could lead to a reduction in the risk of cognitive decline or dementia, without reliable data in this regard [31,32]. 

In our study LVEF (%) was statistically significantly lower in hypertensive patients with AF than in hypertensive patients without AF (*p* < 0.05). The number of patients having LVEF (%)< 50 was five times higher in patients with AF (15 (17.9%) patients) than in those without AF (only 3 (4.2%) patients). Regarding the long-term survival of 8931 patients evaluated in a study on the relationship between AF and LVEF, it was found that the 5-year survival rate was 56% for those with AF compared with 72% for those in sinus rhythm, (*p* < 0.0001). The effect of AF on 5-year survival was most pronounced in those with normal LVEF (62% vs. 78%, *p* < 0.0001) followed by those with a slight reduction in LVEF (57% vs. 72%, *p* = 0.02) [33]. LV dysfunction is associated with an increased risk of AF in men (4.5 times) and women (5.9 times) [34]. The prevalence of AF is related to the extent of LV dysfunction and the patient’s HF status. In patients with pre-existing LV dysfunction, AF may further aggravate chronic heart failure (CHF) symptoms [35]. 

IMT is a noninvasive marker for cardiovascular risk factors and a risk predictor for coronary heart disease (CHD) and stroke [36,37]. IMT is also a marker for highlighting atherosclerosis [38]. In our study, IMT (mm) measured on both CCAs were statistically significantly higher in hypertensive patients with AF than in hypertensive patients without AF, (*p* < 0.05). In a population study, IMT was measured in 4846 subjects in the general population without a history of AF and the association of IMT with the incidence of the first hospitalization of AF over a period of 15.3 years was analyzed. The risk ratio for the incidence of AF was 1.61 (95% (CI): 1.14–2.27). IMT has been independently associated with the incidence of hospitalized AF suggesting that an increase in IMT may predict future AF [39]. In a study published by Kokubo Y. and collaborators in 2018 on the Japanese population, IMT was evaluated and measured at CCA by carotid ultrasonography. Increased IMT and highlighting of carotid atheroma plaques were risk factors for cardiovascular disease (CVD) among the general Japanese population. IMT CCA > 1.1 mm has been shown to be associated with an increased risk of CVD, CHD, and stroke. The new progression of the carotid plaque (IMT > 1.1 mm) was found to be linked to an increased risk of CVD. Carotid ultrasound at CCA is easy to measure as a screening method and thus carotid plaque (CCA IMT > 1.1 mm) could be assessed in order to prevent the increased risk of future CVD [40]. Atherosclerosis and/or IMT can be easily identified without risk and at a low cost and can be incorporated into existing risk scores for AF so as to increase their predictive accuracy. By preventing atherosclerosis and AF, cognitive impairment can also be prevented [41]. In another study performed in young patients without risk factors and heart disease, with AF, AF was significantly associated with carotid-femoral pulse wave speed and IMT CCA [42]. The risk of AF was associated with the severity of carotid plaques (relative risk, 1.49; 95% CI, 1.06–2.10) and with IMT (relative risk, 1.90; 95% CI, 1.20–3.00), in another study. Risk estimates were stronger in women than in men. These outcomes suggest that the correct treatment of asymptomatic atherosclerosis can prevent AF [43]. 

In our study, no statistical changes were observed regarding ABI in both lower limbs, with slightly lower values in hypertensive patients with AF than in hypertensive patients without AF. In a study that evaluated the relationship between AF before and after cardioversion in 99 patients, there were no statistically significant changes in ABI measured before cardioversion and after the restoration of sinus rhythm. ABI right was lower 1.132(1.065–1.210) during AF vs.1.179(1.080–1.242) in sinus rhythm, 95% CI 0.045, *p* = 0.01, and ABI left was lower 1.142 (1.075–1.222) during AF versus 1.170(1.098–1.255) in sinus rhythm, 95% CI 0.040, *p* = 0.011. The results of the study showed that AF had no significant clinical effect on ABI measurements [44]. ABI is considered a strong marker of cardiovascular risk and generalized atherosclerosis [45]. Some data suggest that there may be a direct link between the presence of PAD and an increased risk of AF incidence [46]. The article published by Bekwelem and colleagues presents an analysis derived from the ARIC (Atherosclerosis Risk in Communities) study, about the association between PAD and the risk of new onset of AF, and reported that ABI ≤ 0.90 remained associated with AF incidence after funnel adjustment for race, age, sex, (HR, 1.43; 95%, Cl 1.17–1.75) and borderline ABI (0.91–0.99) is significantly associated with the incidence of AF after adjusting for race, age, sex, (HR, 1.32; 95%, CI 1.16–1.50) [47]. Another meta-analysis suggests that there is a strong relationship between AF and PAD [48]. Another two studies analyzed the relationship between the risk of AF and ABI levels. O’Neal and colleagues conducted an analysis in the MESA (multi-ethnic study of atherosclerosis), a population-based study aimed at assessing the characteristics of subclinical cardiovascular disease and the relationship to risk factors that could predict its progression to clinically obvious disease. In this study, they showed that PAD (defined as both ABI > 1.40 and ABI < 1.00) was associated with incident AF. Also, ABI > 1.40 showed an insignificant trend in association and ABI < 1.0 alone was still found to be associated with the risk of AF [49]. Another observational study looked at the presence of cardiovascular risk factors and pointed out that, there is an important influence on the progression of CVD, especially in subjects over 65 years of age [50]. 

In a study that included patients with amnestic mild cognitive impairment(aMCI) compared to controls, patients with aMCI were significantly older, had higher rates of AF, had a larger IMT, depression, and hypertension [51]. The Atherosclerosis Risk in Communities Neurocognitive Study (ARIC-NCS)analyzed 6432 patients, of whom 611 (9.5%) had AF. AF was associated with dementia (CI 95%, 2.5, 1.64–3.10), respectively, an increased rate of MCI (CI 95%, 1.28, 1.04–1.562). The prevalence of MCI/AD (95% CI, 1.29, 1.04–1.61) and MCI/VD (95% CI, 1.50, 0.99–2.25) was higher in patients with AF than without AF [52]. 

In our study there were 46 (29.7%) patients with diabetes mellitus, 49 (31.6%) were smoking patients, 83 (53.5%) with hyperlipemia, and 25 (16.15) with obesity, these risk factors being present in both groups of patients, their presence increasing the degree of cognitive impairment among those who also associated AF. In ARIC-NCS, patients with AF, diabetes mellitus, lower body mass index, stroke, and older age were linked with the prevalence of dementia [52]. In our study from the AF patients, nine [20%] had minor strokes and 13 [28.9%] had lacunar infarcts. Lower MMSE scores were independently associated with AF, in a study that evaluated patients after ischemic stroke, thus existing with a higher risk of developing MCI (OR 1.6, 95% CI 1.03–2.47, *p* = 0.03) [53]. Even if the patient has associated cerebral vascular pathology or not, the presence of AF increases the risk of stroke, as well as the occurrence of cognitive decline and dementia [54,55]. AF is associated with an increased risk of MCI and dementia, regardless of whether or not patients have a history of stroke, and this is assessed in a meta-analysis [56]. In another meta-analysis that evaluated 27 articles on patients after stroke, it was underlined that age, educational level, type and size of the stroke, hypertension, and diabetes are important risk factors for MCI [57]. The presence of depressive symptoms seems to have an important effect on cognitive impairment. These outcomes are important in order to perform a good strategy after a stroke [57]. 

In a study consisting of 5150 participants, out of whom 552 (10.7%) presented AF, in patients with stroke-free AF, cognitive impairment occurred faster than in patients without AF. There is a higher risk of developing cognitive impairment at younger ages in patients having AF compared to patients without a history of AF [58]. In one study, it was found that in patients over 75 years of age who developed AF compared with those without AF, it was registered a decrease of about three points on the scale of cognitive decline, as age increased by five in 5 years over 75 [58]. The prevalence of AF has been closely associated with MCI and overall impairment of cognitive function, in other studies conducted in Europe and the United States (US) [11,59]. Other studies that have evaluated patients with AF versus patients without AF have shown that there is important proof for the relationship between AF and cognitive decline [58]. 

In our study, MMSE and MoCA decreased statistically significantly in hypertensive patients having AF versus patients without AF (*p* < 0.001).Another study found that stroke-free patients with AF had significantly lower performance in regard to memory, executive function, and learning than those without AF [60]. Several mechanisms have been identified: proinflammatory disorders, micro-lesions, impaired cerebral blood flow (CBF) dynamics, silent cerebral ischemia (SCI), all these being necessary to monitor the association between cognitive decline and AF [61]. 

Depression is linked to a reduction in quality of life, the relationship between depression and MCI being complex [62]. Depression should be recognized and treated as soon as possible and should not be considered normal in the elderly. Regarding cognitive impairment, it is more common in elderly patients with depression and less in young people [12]. Depression influences an individual’s daily activity, it affects their health and their proper functioning [63]. Depression and cognitive impairment are among the DSM criteria for depression [14]. There is a need to establish the diagnosis of depression, to provideoptimal therapy, and to evaluate the results related to long-term depression [12,14]. The symptoms of depression often accompany and influence MCI [12,14]. Depression is often found in cognitive impairment, but also during normal aging [12]. Impaired executive function, information processing speed, amnestic and attentional functions are common in patients with depression [13]. Symptoms of depression can also occur before cognitive impairment [13]. Depression influences the depletion of cognitive reserve and it aggravates the pre-existing cognitive impairment [12]. Signs of depression may be signs of neurodegeneration, and these previous manifestations may influence the evolution of cognitive and functional manifestations [12]. Functional impairment is a mandatory feature of MCI and it partially depends on how severe the cognitive impairment is [12]. Functional capacity is more associated with depression [12]. Depression can influence the course of cognitive impairment; it affects the balance of these patients and thus increases the risk of falls [12]. These patients have higher rates of depression compared to healthy people, regardless of gender, race, age, and type of study [12].Depression can lead to physical, cognitive, or social disorders if it is not treated in time [63]. With the aging population, depression is more common, accounting for about 13% of the elderly population. Depression was found in 43% of patients requiring home care and in 24% of elderly patients with associated medical pathology [64]. Depression was highlighted as a risk factor for MCI in a meta-analysis [65], while other studies, found an association between depression and MCI with prevalence but not incidence. These analyses suggest that depression accompanies MCI, but does not precede it [66]. 

ADL significantly decreased in patients with paroxysmal AF, compared with patients with persistent or permanent AF (*p* < 0.05), in our study. The efficiency of the ADL scale is higher when patients have more severe cognitive impairment and dementia, and the efficiency of the IADL scale is sensitive even when patients present with MCI [64]. In our study, patients with permanent or persistent AF were double compared to patients with paroxysmal AF. We recorded no differences between other vascular parameters or neuropsychological parameters in patients with different types of AF. The ARIC (US-based Atherosclerosis Risk in Communities) study found that persistent AF is associated with impaired cognitive function, while patients with paroxysmal AF were not affected. Thus, it was highlighted that the presence of AF over time can lead to cognitive disorders [67]. If patients with AF have a higher risk of cognitive impairment, it is very important to evaluate all factors that cause cognitive impairment, to intervene and prevent complications of cognitive impairment in patients with this arrhythmia [60]. The occurrence of AF was associated by lowering the MMSE score with the faster presence of cognitive impairment, in a study that followed patients for a period of 7 years [58]. 

Currently, there are insufficient data to highlight whether inadequate anticoagulation could be involved in the development of cognitive impairment. In one study, patients treated with OACs (AVK) who did not have an optimal therapeutic interval had a higher risk of developing dementia. At this time there is no clear evidence on the effect of anticoagulant treatment on the development of cognitive disorders; clinical studies that are ongoing consider the effectiveness and improvement of anticoagulation quality [68]. In our study, IMT increased and left ventricular parameters decreased, but were not statistically significant in patients with OACs (AVK) versus NOACs therapy. We observed a lower risk of cognitive impairment in patients receiving NOACs than those receiving warfarin, issues highlighted in a recent study on the risk of dementia in patients diagnosed with AF who received anticoagulant therapy [68,69]. The association between MCI and AF can be explained due to the presence of lacunar infarcts, cerebral micro-bleeding that can lead to cerebral hemorrhages, and cerebral neurodegenerative changes [70]. One study found that the risk of micro-bleeding was higher among patients who showed greater variability in anticoagulation control, and patients using AVK had a higher incidence and prevalence of micro-bleeding [71]. 

Ofall patients with AF, 63 (75%) had a CHA₂DS₂-VASc score > 3 and 21 (25%) patients had a CHA₂DS₂-VASc score < 3. MoCA and MMSE significantly decreased in patients with CHA2DS2-VASc >3 and compared with patients with CHA2DS2-VASc <3 (*p*< 0.05). ADL, IADL, and GDS-15 significantly increased in patients withCHA2DS2-VASc > 3 and AF compared with patients with CHA2DS2-VASc <3 and AF (*p* < 0.05). In patients with AF, adequate control of the risk factors included in the CHA2DS2-VASc score is needed to prevent the occurrence or exacerbation of cognitive impairment. CHA2DS2-VASc, HAS-BLED, HR, male sex, higher LV mass index, decreased renal function, and troponin T were significantly linked to the occurrence of a higher number of MCI disorders or dementia in the ARIC-NCS study [52]. It has been found that, in order to prevent cognitive impairment, anticoagulation regimens should be carefully considered as the CHA2DS2-VASc score is higher [69]. Older age is an important component in calculating the CHA2DS2-VASc score but also an important risk factor for cognitive impairment [11]. The Rotterdam study showed that there is a double risk of cognitive impairment in patients with AF than those without AF, this risk being higher in those under 65 years and more common in females [72]. 

It is very important to periodically evaluate patients with cardiovascular risk factors present, especially to monitor the occurrence and presence of AF in hypertensive patients with other cardiovascular risk factors present, by laboratory tests and periodic hemodynamic parameters (SBP, DBP, HR), by analysis of echocardiographic parameters, to detect any decrease in LVEF and, thus, of signs of heart failure, by IMT analysis at CCA level to detect any increase in this parameter and, thus, the occurrence of atherosclerosis, stroke risk, and ischemic coronary heart disease as well as ABI analysis by which any decrease in this parameter could lead to PAD.

A weakness of the study was that cranial CT could not be performed for the patients included in the study.

Blood pressure control, intervention on endothelial dysfunction, left atrial thrombotic risk and cerebral thromboembolism are, included in our study, coherent and feasible objectives, given the frequent follow-up and the monitoring of patients at high risk of accelerated cognitive degradation.

Also, periodic evaluation of cognitive tests, tests to assess signs of depression, and analysis of the patient’sdaily activity, correlated with paraclinical explorations to highlight hemodynamic, laboratory, and subclinical atherosclerosis parameters, could more quickly detect signs of cognitive impairment as well as avoid the occurrence of cardiovascular and cerebrovascular events. Thus, through a multidisciplinary clinical approach and treatment, interventions could occur more quickly in order not to reach an unfavorable evolution of patients in terms of memory disorders, depression, and daily activity, an aspect that represents a significant long-term psychosocial impact.

Studies in the literature claim that AF is an important risk factor for cognitive impairment. The mechanisms responsible for this association are diverse and are associated with the well-established increase in the risk of stroke in people with AF. Future research needs to better analyze these mechanisms and to develop clinical and therapeutic interventions that reduce the risk of cognitive impairment associated with AF and other risk factors [11]. 

## 5. Conclusions

The existence of cognitive impairment has a very important psychosocial impact on the population, so it is very important to carefully evaluate cardiovascular risk factors and associated pathologies, hypertension, and AF, being two very important cardiovascular risk factors in the occurrence of cardiovascular and cerebrovascular events. Where hypertensive patients, regardless of its degree of impairment, have associated AF, regardless of its type (paroxysmal, persistent, or permanent), the risk of cognitive impairment and depression increases, the ability to manage daily activity decreases, and the bigger risk of occurrence of subclinical atherosclerosis increases, all these being correlated with older age, the type of anticoagulant used and the CHA₂DS₂-VASc score, thus there is a higher risk of occurrence of cerebrovascular events.

The better we manage to highlight and control hypertensive patients where there is an associated AF in terms of cognitive impairment and subclinical atherosclerosis, by properly managing treatment and assessing the risk of vascular events, the better we can prevent stroke and dementia. Moreover, our study also showed that in most situations, the therapeutic target in blood pressure control should be lowered to lower values than in the indications of the ESC 2018 guide, even in patients over 65 years for correct prevention of cognitive decline, not only the risk of AF and thromboembolism.

Our study revealed that it is particularly important where we have a patient with hypertension and associated AF, to assess the degree of cognitive impairment using screening tools for cognitive impairment, daily activity and depression scales, and based on these and other clinical and paraclinical criteria to establish appropriate therapeutic behavior. If our patient has a higher CHA₂DS₂-VASc score and is aged above 65, we can better know the patient’s risk of stroke and we can use neurocognitive assessment tests and subclinical atherosclerosis assessment parameters faster, to prevent cognitive impairment and possible cardiovascular or cerebrovascular events.Both the risk and the therapeutic conduct should obviously be individualized, and the specific blood pressure, lipid, diabetic, vascular control molecules should be guided by this aspect. Of interest is also the integration of aspects of vascular atherosclerosis with other locations, which leads to a more aggressive therapeutic attitude.

The association of the periodic individual assessment of cognitive capacity with that of risk parameters-atherosclerotic, blood pressure, arrhythmic, thrombotic/hemorrhagic can lead to saving quality and life expectancy, especially in patients over 65 years of age, knowing that uncontrolled hypertension in medium-age adults leads to significant vascular and cerebral damage. Of course, primary prevention measures, instituted as early as possible, are essential. Moreover, the new magnitude taken by DNA sequencing can detect the determined genetic risk, not to be neglected in individualized modern medicine.

Future studies will be able to highlight, with greater precision, the strategy to manage cognitive impairment, daily activity, cardiovascular risk factors, treatment used, and risk scores regarding early detection and reduction, in number, of patients with cognitive impairment and associated cardiovascular and cerebrovascular pathology.

## Figures and Tables

**Table 1:** Patient demographic data, cardiovascular risk factors data.

Patients Demographic Data	All Patients Included in the Study	84 Hypertensive Patients with AF	71 Hypertensive Patients without AF
	Number of patients/% Mean ± SD	Number of patients/% Mean ± SD	Number of patients/% Mean ± SD
Age	155/69.98 ± 9.89	84/54.2/73.88 ± 9.78	71/45.80/68.00 ± 9.23
Age < 65	45/29.0/59.38 ± 5.78	18/21.4/59.61 ± 5.82	27/38.0/59.22 ± 5.86
Age > 65	110/71.0/76.02 ± 6.72	66/78.6/77.77 ± 6.45	44/62.0/73.39 ± 72.0
Sex/Male/Age	72/46.5/69.82 ± 10.00	36/42.9/72.61 ± 10.08	36/50.7/67.03 ± 9.24
Sex/Female/Age	83/53.5/72.37 ± 9.80	48/57.1/74.83 ± 9.54	35/49.3/69.00 ± 9.25
Diabetes mellitus	46/29.7	21/25.0	25/35.2
Smoking	49/31.6	24/28.6	25/35.2
Hyperlipemia	83/53.5	42/50.0	41/57.7
Obesity	25/16.1	10/11.9	15/21.1
Chronic kidney disease	38/24.5	26/31.0	12/16.9
Peripheral arterial disease	10/6.5	4/4.6	6/8.5
Coronary disease	68/43.9	38/45.2	30/42.3
Heart failure	68/43.9	41/48.8	27/38.0
Lacunar stroke	51/32.9	26/31.0	25/35.2
Minor stroke	22/14.2	15/17.9	7/9.9

SD: Standard Deviation.

**Table 2:** Biological parameters in hypertensive patients with AF and hypertensive patients without AF.

Biologic Parameter	All Patients		84 Hypertensive Patients with AF		71 Hypertensive Patients without AF		*p*-Value *
	Mean	SD	Mean	SD	Mean	SD	
Hemoglobin (g/dL)	13.85	1.94	13.57	2.07	14.18	1.72	0.049
Hematocrit (%)	41.47	5.18	40.78	5.69	2.29	4.42	0.066
ESR (mm/h)	22.03	15.89	21.75	16.09	22.38	15,76	0.807
Thrombocytes x10^3^/µL	227.29	58.90	222.83	60.15	232.56	57.36	0.037
APTT(s)	27.74	6.70	28.87	7.97	26.41	4.46	0.017
INR	1.95	0.78	1.86	0.86	2.05	0.68	0.135
PT (s)	24.63	10.69	24.17	11.09	25.17	10.26	0.564
PT (%)	57.46	29.43	60.72	29.87	53.60	28.63	0.134
Total Cholesterol (mg/dL)	183.86	60.48	170.17	54.50	200.05	63.51	0.002
LDL Cholesterol (mg/dL)	131.30	52.52	121.30	47.06	143.14	56.39	0.011
HDL Cholesterol (mg/dL)	47.98	18.14	45.90	12.06	50.43	23.24	0.141
TG (mg/dL)I	130.91	56.98	123.29	47.62	139.92	65.58	0.078
Glycemia (mg/dL)	110.02	30.34	109.25	35.68	110.94	22.67	0.730
Urea (mg/dL)	41.85	17.93	43.58	20.97	39.80	13.33	0.176
Creatinine (mg/dL)	0.98	0.24	1.04	0.23	0.91	0.23	0.001
ALT (U/L)	29.89	20.74	32.77	26.23	26.49	10.44	0.046
AST (U/L)	26.05	17.80	29.22	22.91	22.30	6.97	0.010
CKMB (U/L)	17.03	8.98	18.59	11.02	15.18	5.22	0.013
K (mmol/L)	4.11	0.43	4.05	0.48	4.18	0.35	0.065
Na (mmol/L)	140.77	2.58	140.87	2.64	140.64	2.51	0.583
Uric acid (mg/dL)	6.26	1.56	6.38	1.64	6.11	1.45	0.280

ESR: erythrocytes sedimentation rate; APTT: activated partial thromboplastin time; INR: international normalized ratio; PT: prothrombine time; LDL: low-density lipoprotein; HDL:high-density lipoprotein; TG: triglycerides; ALT:alanine aminotransferase; AST: aspartate aminotransferase; CKMB: creatine kinase MB; K: potassium; Na: sodium.* *p*-values obtained with unpaired t-tests applied independently for each variable to test for differences between the two groups of patients (hypertensive patients with AF and hypertensive patients without AF).

**Table 3:** Hemodynamic parameters, IMT, and ABI in hypertensive patients with/without AF.

Hemodynamic Parameters	All Patients		84 Hypertensive Patients with AF		71 Hypertensive Patients without AF		*p*-Value*
			Mean	SD	Mean	SD	
SBP (mmHg)	132.06	19.97	136.31	22.61	127.04	14.96	0.003
DBP (mmHg)	78.16	14.32	80.71	14.16	75.14	14.01	0.015
HR (b/min)	74.93	15.44	76.68	17.50	72.86	12.38	0.115
IMT (mm)left	0.69	0.27	0.75	0.27	0.63	0.26	0.005
IMT (mm) right	0.67	0.30	0.75	0.32	0.59	0.26	0.001
ABI left	1.10	0.14	1.09	0.10	1.11	0.17	0.434
ABI right	1.10	0.14	1.09	0.16	1.08	0.12	0.625

SBP: systolic blood pressure; DBP: diastolic blood pressure; HR: heart rate; IMT: intima–media thickness; ABI: ankle brachial index.* *p*-values obtained with unpaired *t*-tests applied independently for each variable to test for differences between the two groups of patients (hypertensive patients with AF and hypertensive patients without AF).

**Table 4:** Echocardiographic parameters of left ventricle systolic function in both groups of patients. Assessment of diastolic transmitral flow and LV systolic function using pulsed wave Tissue Doppler Imaging at the lateral mitral annulus.

Echocardiographic Parameters	All Patients		84 Hypertensive Patients with AF		71 Hypertensive Patients without AF		*p*-Value *
	Mean	SD	Mean	SD	Mean	SD	
IVS (mm)	11.99	2.11	12.26	1.90	11.68	2.31	0.087
LA (mm)	41.37	6.25	43.25	6.50	39.15	5.15	<0.001
LVPW (mm)	12.08	2.50	11.95	2.34	12.24	2.70	0.509
LVESD (mm)	24.29	6.67	23.96	7.18	24.67	6.04	0.510
LVEDD (mm)	46.51	5.33	47.83	5.84	44.95	4.19	0.001
LVESV (mL)	35.83	13.81	37.76	15.02	33.56	11.94	0.059
LVEDV (mL)	76.75	19.28	78.35	19.88	74.85	18.51	0.262
LVEF (%)	57.51	6.92	56.29	7.77	58.95	5.45	0.014
E (m/s)	0.71	0.18	0.74	11.820.19	0.69	0.17	0.084
PSAP (mmHg)	39.13	11.66	42.23		35.46	10.41	<0.001

IVS: interventricular septum; LA: left atrial; LVPW: left ventricular posterior wall; LVESD: left ventricular end-systolic diameter; LVEDD: left ventricular end-diastolic diameter; LVESV: left ventricular end-systolic volume; LVEDV: left ventricular end-diastolic volume; LVEF: left ventricular ejection fraction; E: peak early diastolic transmitral flow velocity; PSAP: pulmonary systolic arterial pressure.* *p*-values obtained with unpaired t-tests applied independently for each variable to test for differences between the two groups of patients (hypertensive patients with AF and hypertensive patients without AF).

**Table 5:** Screening tools for cognitive impairment, daily activity, and depression scalesin both groups of patients.

Screening Tools for Cognitive Impairment, Daily Activity and Depression Scales	All Patients		84 Hypertensive Patients with AF		71 Hypertensive Patients without AF		*p*-Value*
	Mean	SD	Mean	SD	Mean	SD	
MMSE	26.29	4.47	25.15	4.92	27.63	3.46	<0.001
MoCA	24.35	5.37	23.44	5.50	25.42	5.04	0.022
ADL	9.42	1.03	9.69	0.78	9.19	1.18	0.002
IADL	6.72	1.77	7.18	1.34	6.33	1.99	0.002
GDS-15	6.65	2.30	7.08	2.29	6.15	2.23	0.012

MoCA: Montreal Cognitive Assessment Scale; MMSE: Mini-Mental State Examination Scale; IADL: Instrumental Activities of Daily Living Score; ADL: Activities of Daily Living Score; GDS-15: Geriatric Depression Scale 15 questions.* *p*-values obtained with unpaired t-tests applied independently for each variable to test for differences between the two groups of patients (hypertensive patients with AF and hypertensive patients without AF).

**Table 6:** Screening tools for cognitive impairment, daily activity, and depression scalesin hypertensive and AF patients with score CHA₂DS₂-VASc <3 versus CHA₂DS₂-VASc >3.

Hypertensive and AF Patients	Patients with CHA₂DS₂-VASc <3	Patients with CHA₂DS₂-VASc >3	*p*
Screening tools for cognitive impairment, daily activity, and depression scales	Mean ± SD	Mean ± SD	
MMSE	27.81 ± 2.04	24.27 ± 5.28	<0.001
MoCA	26.81 ± 2.78	22.32 ± 5.73	<0.001
ADL	8.95 ± 1.27	9.90 ± 0.30	0.001
IADL	6.08 ± 2.16	7.10 ± 1.04	0.006
GDS-15	5.76 ± 2.02	7.52 ± 2.22	0.002

MoCA: Montreal Cognitive Assessment Scale; MMSE: Mini-Mental State Examination Scale; IADL: Instrumental Activities of Daily Living Score; ADL: Activities of Daily Living Score; GDS-15: Geriatric Depression Scale 15 questions.

**Table 7:** IMT and screening tools for cognitive impairment, daily activity, and depression scalesin both groups of patients adjusted for age and sex.

Variable Analyzed Adjusted for Age and Sex	84 Hypertensive Patients with AF		71 Hypertensive Patients without AF		*p*-Value*
	Mean	Std.Error	Mean	Std. Error	
IMT (mm) left adjusted for age IMT (mm) left adjusted for sex	0.740 0.750	0.030 0.030	0.650 0.630	0.030 0.030	0.009 0.842
IMT (mm) right adjusted for age IMT (mm) right adjusted for sex	0.730 0.750	0.030 0.030	0.610 0.590	0.030 0.040	0.015 0.205
MMSE adjusted for age MMSE adjusted for sex	25.567 25.132	0.455 0.472	27.147 27.661	0.496 0.472	<0.001 0.363
MoCA adjusted for age MoCA adjusted for sex	23.764 23.403	0.578 0.578	25.040 25.467	0.631 0.629	0.007 0.223
ADL adjusted for age ADL adjusted for sex	9.602 9.691	0.119 0.120	9.265 9.190	0.109 0.111	0.001 0.911
IADL adjusted for age IADL adjusted for sex	7.036 7.202	0.204 0.204	6.458 6.318	0.187 0.188	0.001 0.121
GDS-15 adjusted for age GDS-15 adjusted for sex	6.873 7.095	0.239 0.248	6.404 6.141	0.261 0.270	<0.001 0.378

IMT: intima–media thickness; MoCA: Montreal Cognitive Assessment Scale; MMSE: Mini-Mental State Examination Scale; IADL: Instrumental Activities of Daily Living Score; ADL: Activities of Daily Living Score; GDS-15: Geriatric Depression Scale 15 questions.*p-values obtained with ANCOVA test with control for age and sex.

## Data Availability

The data analyzed during the current study are available from the corresponding author on reasonable request.
